# Targeting disrupted networks in schizophrenia: Can muscarinic drugs make a fundamental difference?

**DOI:** 10.1177/02698811251375104

**Published:** 2025-10-28

**Authors:** Judith A. Pratt, Brian J. Morris

**Affiliations:** 1 Strathclyde Institute of Pharmacy and Biomedical Science, University of Strathclyde, Glasgow, UK; 2 College of Medical Veterinary and Life Sciences, School of Psychology and Neuroscience, University of Glasgow, UK

**Keywords:** dopamine receptor antagonist, M1 receptor, M4 receptor, M2 receptor, M5 receptor, thalamus, 5-HT1A receptor, 5-HT7 receptor, transdiagnostic

## Abstract

New treatments for schizophrenia are urgently needed because existing antipsychotic drugs mainly improve positive symptoms, with minimal effect on cognitive deficits and negative symptoms. The approval of Karuna Therapeutics (KarXT) in 2024 marked a significant milestone, as it became the first antipsychotic drug to target muscarinic acetylcholine receptors (mAChRs) rather than dopamine receptors. Here, we provide a perspective on how targeting mAChRs might improve the positive, negative and cognitive symptoms of schizophrenia. First, we revisit the prevailing view that xanomeline acts primarily as a M1 and M4 mAChR partial agonist. Next, we examine potential pharmacological overlap with clozapine, focusing on actions at 5-HT1A, 5-HT2A and 5-HT7 receptors and consider whether 5-HT receptor subtype agonism, inverse agonism or antagonism could be important for therapeutic efficacy. We then review the brain systems and networks impacted by muscarinic receptor subtypes in the context of Research Domain Criteria (RDoC) domains. We propose that, based on their cellular and regional expression, muscarinic receptor subtypes impact several cortico-striatal-thalamo-cortical loops and interrelated networks to improve RDoC-informed sensorimotor, positive valence, social processes, arousal and regulation, and cognitive systems. Taken together, these data suggest that there are neurobiological reasons for optimism for muscarinic agents to improve the classically described positive, negative and cognitive symptoms of schizophrenia, although the relative contribution of each mAChR subtype (M1–M5) remains unclear. We propose that a multi-targeted approach combining actions at 5-HT1A and 5-HT7 receptors could provide additional therapeutic benefits across a range of RDoC domains and hence be of clinical benefit trans-diagonistically beyond schizophrenia.

## Introduction and context

The dawn of a new era in schizophrenia treatment may have begun in September 2024 when the US Food and Drug Administration (FDA) approved the drug Karuna Therapeutics (KarXT) (Cobenfy; Dolgin, 2024). KarXT is the first antipsychotic drug to target muscarinic receptors rather than dopamine receptors. However, the idea that muscarinic receptor agonists could be useful for treating schizophrenia is not new. Clinical reports from the 1950s suggested that arecoline, a plant alkaloid found in betel nut, could improve schizophrenia symptoms. Later, in the 1980s and 1990s, when muscarinic agonists were being evaluated for improving cognition in Alzheimer’s disease, researchers observed that xanomeline unexpectedly produced striking improvements in psychotic and behavioural symptoms in patients. Subsequently, xanomeline showed promise in schizophrenia for treating psychotic symptoms and improving cognition (Bodick et al., 1997). However, its development was halted due to peripherally mediated side effects. A key breakthrough in the development of KarXT (Cobenfy) was combining xanomeline with a peripherally acting muscarinic receptor antagonist (trospium chloride), which mitigated these side effects, allowing the drug to move forward in development.

A key question is whether or not muscarinic receptor agonists will mark a fundamental shift in the treatment of schizophrenia, taking over from existing antipsychotic medications that target dopamine receptors. The location of muscarinic receptors in brain networks altered in schizophrenia is key to understanding how drugs such as xanomeline could improve symptoms. For decades, the focus has been on antipsychotic drugs targeting disruption of the midbrain dopamine system in relation to positive (psychotic) symptoms, although this is now considered an oversimplification. Existing drugs have limited impact on cognitive deficits and negative symptoms, which are important predictors of long-term functional outcome and quality of life. Hence, understanding the networks involved in cognitive function and negative symptoms is important for determining whether treatments can address these wider dysfunctions.

Current diagnosis of schizophrenia is based on symptom clusters (Diagnostic and Statistical Manual of Mental Disorders, Fifth Edition (DSM-5) and International Classification of Diseases, 11th Revision (ICD-11)) rather than underlying changes in neurobiology and behaviour. The Research Domain Criteria (RDoC) project (https://www.nimh.nih.gov/research/research-funded-by-nimh/rdoc) has emerged as a translational framework to develop new ways of classifying mental health conditions based upon neurobiological and behavioural components that may cross existing diagnostic categories. RDoC organises neuro-behavioural processes into six domains: positive valence systems, negative valence systems, cognitive systems, sensorimotor systems, systems for social processes and arousal and regulatory systems. Each domain consists of constructs that can be studied at multiple levels, from genes and molecules to cells, circuits, physiology, behaviour and self-report. This approach facilitates a more precise and personalised treatment strategy, moving beyond broad diagnostic categories to target specific symptom domains across multiple conditions (i.e. transdiagnostic).

By framing schizophrenia within an RDoC-informed model, treatment strategies such as muscarinic receptor modulation can be more directly linked to the underlying neurobiological mechanisms driving symptoms.

Here, we provide a perspective on

- The pharmacological profile of xanomeline and comparison with existing antipsychotic drugs
- The impact of muscarinic receptors in brain regions and circuits implicated in schizophrenia in the context of RDoC domains and constructs.
- Whether muscarinic agonism alone is sufficient to improve schizophrenia symptoms, informed by RDoC.

We propose an optimal path forward incorporating additional targets that may provide a synergistic role in restoring disrupted neural circuits and alleviating symptoms.

## Pharmacological profile of xanomeline and comparison with existing antipsychotic drugs

### Preclinical pharmacology

Muscarinic acetylcholine receptors (mAChRs) mediate the physiological actions of the endogenous neurotransmitter acetyl choline (ACh) and are composed of five distinct subtypes, M1–M5. They are all G-protein-coupled receptors. M1, M3 and M5 receptors are stimulatory and are coupled to Gaq/11 proteins and stimulation of phospholipase C, while M2 and M4 receptors are inhibitory and are coupled primarily to Gai/o proteins and inhibition of adenyl cyclase. All five mAChRs are expressed throughout the body and have distinct expression levels in the central nervous system (CNS). Development of selective orthosteric ligands for muscarinic receptors has proved challenging since the mAChR family is amongst the most highly conserved in terms of protein structure and sequence (Paul et al., 2024).

The prevailing impression is that xanomeline acts primarily as a partial agonist at M1 and M4 muscarinic cholinergic receptors. The evidence for this derived originally from experiments on peripheral tissues, where a much clearer partial agonist action was detected in rabbit vas deferens (M1 receptors) as compared to guinea-pig atria (M2 receptors) and guinea-pig ileum (probably M3 receptors; Shannon et al., 1994). However, xanomeline did also show some slight partial agonist activity in guinea-pig bladder (M3 receptors; Shannon et al., 1994). The precise level of partial agonist activity in the brain is difficult to assess – it depends on the receptor reserve at that location, and will almost certainly differ from that in peripheral tissues, in fact probably varying from region to region in the brain, and from neuron to neuron, and even between subcellular compartments (i.e. presynaptic terminal vs dendrite).

It is clear that xanomeline has high affinity for all muscarinic receptors (M1–M5), and the affinity is similar for all (Wood et al., 1999; Powers et al., 2023; Watson et al., 1998). Thus, xanomeline will clearly act at central M2, M3 and M5 receptors as well as M1 and M4; it is just not clear whether the interaction is as an antagonist or as a weak (or strong) partial agonist. Some in vitro functional systems (transfected cells) have very high receptor reserve and facilitate the demonstration of partial agonist activity. Indeed, in vitro, xanomeline appears as a partial to full agonist at all muscarinic receptors (M1–M5; Davies et al., 2005; Grant and El-Fakahany, 2005; Heinrich et al., 2009; Messer et al., 1997; Noetzel et al., 2009; Odagaki et al., 2016; Olianas et al., 1999; Powers et al., 2023; Watson et al., 1998; Wood et al., 1999).

Partial agonist activity at M1 and M4 receptors has been observed in CNS tissue, in rodent frontal cortex, hippocampus and striatum (Odagaki et al., 2016; Thorn et al., 2019). Effects on striatal dopamine release originally interpreted as M1 agonist actions (Shannon et al., 1994) may, in the light of current knowledge, be more plausibly explained by M5 agonist actions (Razidlo et al., 2022; Zell et al., 2023). The level of partial agonist activity that might exist at the other muscarinic receptors appears not to have been systematically investigated and remains unclear. However, in addition to M1/M4 (and possibly M5) receptor partial agonist actions, xanomeline shows partial agonist at 5-HT1A and 5-HT2A serotonin receptors, and this is seen both in vitro and in brain (cortex and hippocampus) tissue (Davies et al., 2005; Heinrich et al., 2009; Odagaki et al., 2016; Watson et al., 1998). As the potency of these serotonergic effects is equivalent to the muscarinic receptor potency, these actions are likely to form a part of the clinical picture of xanomeline.

Xanomeline does show some very weak affinity for D2 dopamine receptors, but it is probably sufficiently weak not to be relevant at therapeutic doses (Watson et al., 1998).

The profile of partial agonist activity at M1 and M4 muscarinic receptors, and at 5-HT1A receptors, corresponds quite closely to the pharmacological profile of clozapine. Clozapine shares high affinity for all muscarinic receptors, with partial agonist activity, at least in vitro, in each case (Michal et al., 1999; Olianas et al., 1997, 1999; Zorn et al., 1994), combined with partial agonist activity at 5-HT1A receptors (Newman-Tancredi et al., 1996). There are some differences: apart from clozapine’s significant D2 dopamine receptor activity, clozapine is an antagonist rather than a partial agonist at 5-HT2A receptors, and also has inverse agonist activity at 5-HT7 receptors (Krobert and Levy, 2002; Mahé et al., 2004) (where xanomeline has affinity, but unknown efficacy) Myslivecek, 2022; Watson et al., 1998). Clozapine also has a number of other actions as well, for example, at histamine H1 receptors.

In summary, it is possible that xanomeline’s partial agonist actions in the CNS are limited to M1, M4, 5-HT1A and 5-HT2A receptors, although it will also bind to the other muscarinic receptors, where its efficacy is not totally clear. This pattern of receptor interaction shares some similarities with that of clozapine with respect to the muscarinic and 5-HT1A receptor actions.

Emraclidine is a positive allosteric modulator at M4 muscarinic receptors. Comparing emraclidine with xanomeline, the former appears to be more selective, acting mainly at M4 receptors, and to a much lesser degree at M2 receptors. There seems to be no relevant affinity for other muscarinic receptors (Butler et al., 2024), but possible serotonergic actions appear not to have been investigated.

## Impact of muscarinic receptors in neural systems and circuits implicated in schizophrenia in the context of RDoC domains

Table 1 summarises the neural systems implicated in schizophrenia from an RDoC perspective. Notably, for many RDoC domains, regions of the corticostriato-thalamo-cortical loop (CSTC) circuits, amygdala and hippocampus are recruited (See Table 1). Separate CSTC loops have been associated with different behaviours, including sensorimotor, associative (cognitive) and limbic (affective/motivational); each engaging specific regions of the cerebral cortex, striatum and thalamus (Alexander et al., 1986, 1990). Initially, these loops were considered to function as parallel segregated circuits, although accumulating evidence shows that projections from functionally diverse cortical regions overlap within the striatum and that hub regions exist that integrate and communicate information across functional systems (Haber et al., 2006, 2016).

**Table 1.** Overview of brain systems implicated in schizophrenia in the context of RDoC domains.

Symptom domain	Key symptoms	RDoC domain	Brain systems	Construct dysfunction	Reference
Positive (psychotic symptoms)	Hallucinations (mainly auditory)	Sensorimotor (perception)	Auditory cortex, medial geniculate, superior temporal gyrus	Impaired sensory filtering leading to sensory overload and abnormal perceptions	Ford et al. (2014)
		Sensorimotor (perception)	Insular cortex	Impaired self-monitoring; internally generated speech and thoughts misattributed to external	Wylie and Tregellas (2010)
	Delusions	Positive valence system	Mid-brain dopamine system	Aberrant salience-neutral stimuli take on excessive significance	Kapur (2003), Howes et al. (2020), Kesby et al. (2023), but see Corlett and Fraser (2025)
		Cognitive systems	PFC, ACC, striatum, hippocampus	Prediction error mismatch between expectation and experience- assignment of meaning to irrelevant events, reinforcing false beliefs	Corlett and Fraser (2025), Corlett et al. (2010)
	Disordered thinking and speech	Cognitive systems	PFC, thalamus, STG (incl Wernicke’s area)	Impaired working memory, logical sequencing, speech production and language processing	Chang et al. (2022)
Negative symptoms	Avolition (amotivation)	Positive valence systems	SN, VTA, dorsal and ventral striatum, DLPFC, ACC, OFC	Reduced effort-based decision-making and reward prediction	Strauss et al. (2014), Barch et al. (2016), Dexter et al. (2025)
	Anhedonia	Positive valence systems	SN, VTA, dorsal and ventral striatum, DLPFC, ACC, OFC	Impaired reward sensitivity and learning	Barch et al. (2016), Dexter et al. (2025)
	Blunted affect	Arousal and regulation	Amygdala, VL PFC, ACC, Basal ganglia	Reduced emotional expression and processing	Barch et al. (2016), Dexter et al. (2025), Bègue et al. (2020)
	Asociality	Social processes	mPFC, ACC, amygdala, STS	Social withdrawal-impaired social cognition	Bersani et al. (2014)
Cognitive	Working memory	Cognitive systems	DLPFC, mediodorsal thalamus, hippocampus, parietal córtex	Impairments in holding, retrieving and manipulating information	Smucny et al. (2022), Bolkan et al. (2017)
	Executive function	Cognitive systems	DLPFC, OFC, ACC, striatum	Deficits in planning, problem-solving and cognitive flexibility	Smucny et al. (2022)
	Attention	Cognitive systems	Parietal cortex, dACC, DLPFC, parietal cortex, thalamic pulvinar nucleus	Deficits in selective and sustained attention and filtering distractions	Smucny et al. (2022)

ACC: anterior cingulate cortex; OFC: orbitofrontal cortex; PFC: prefrontal cortex; SN: substantia nigra; STS: superior temporal sulcus; STG: superior temporal gyrus; VTA: ventral tegmental area; DLPFC: dorsolateral prefrontal cortex; VLPFC: ventrolateral prefrontal cortex.

*Positive symptoms* of schizophrenia include hallucinations (typically auditory), delusions and disordered speech and thinking. From an RDoC perspective, positive symptoms can be understood through multiple domains: positive valence system, cognitive systems, social processes and sensorimotor systems (Table 1).

Auditory hallucinations (sensorimotor RDoC domain) are related to impaired thalamo-cortical sensory processing in the auditory cortex, inferior colliculus and medial geniculate of thalamus (Ford et al., 2014) together with recruitment of a wider network of regions, including the superior temporal gyrus and anterior insula (Ford et al., 2014). The perception of auditory hallucinations involves RDoC perceptual domains. For example, the insula cortex is important in interoception and self-awareness (Gogolla, 2017; Wylie and Tregellas, 2010), and the insula (together with the anterior cingulate cortex) may misattribute internally generated speech as coming from an external source, resulting in ‘hearing voices’.

Delusions are strong false beliefs and come in many forms, including paranoia, feeling controlled by an outside force, and thinking one is famous. The neurobiological processes remain incompletely understood. One highly cited theory is that delusional thinking results from aberrant salience (Kapur, 2003), where neutral or random stimuli are mistakenly perceived as meaningful or significant. The suggestion is that mesolimbic dopamine dysfunction relates to incentive salience and could explain psychotic symptoms. As such, this relates to the ‘positive valence’ RDoC domain (Dexter et al., 2025). The neurobiological and behavioural basis of the incentive salience theory has been challenged, and a prominent theory based upon animal learning and Bayesian inference (see Corlett et al., 2010; Ermakova et al., 2018; Millard et al., 2022) suggests that delusions arise from errors in how the brain processes predictions and uncertainty (Corlett and Fraser, 2025). These prediction error deficits align with RDoC cognitive domains.

Whilst striatal dopamine dysfunction has been associated with prediction error signalling and aberrant salience (Howes et al., 2020), it is important to consider this in the context of interconnected neural networks recruiting dorsolateral prefrontal cortex (DLPFC), hippocampus and amygdala, and their underlying neurotransmitter systems (Corlett and Fraser, 2025; Lisman and Grace, 2005). For example, the prediction error signals from the dopaminergic ventral tegmental area (VTA) involve the interplay between prefrontal cortex regions, particularly the anterior cingulate cortex and orbitofrontal cortex (Behrens et al., 2009; Kesby et al., 2023; Soltani and Wang, 2010; Takahashi et al., 2009; see Corlett et al., (2010)). Importantly, recent evidence demonstrates that dopamine dysfunction is altered in the associative striatum rather than the limbic striatum, which is reciprocally connected to the DLPFC (Corlett et al., 2010; Haber et al., 2006; McCutcheon et al., 2018). Taken together, this aligns with ‘prediction error’ deficits falling under RDoC cognitive systems.

## Negative symptoms and cognitive deficits

From a RDoC perspective, negative symptoms of avolition and anhedonia fall under the ‘positive valence system’ domain, blunted affect under ‘arousal and regulation’ and asociality under ‘social processes’ domain. Cognitive deficits fall under RDoC ‘cognitive systems’ domain and include working memory, executive function, attention and processing speed components (Barch and Young, 2023) (See Table 1).

## Muscarinic receptor localisation in neural systems informed by RDoC

Cholinergic neurones from the brainstem and the basal forebrain regions project widely to regions such as the thalamus, basal ganglia, hippocampus, anterior cingulate, amygdala and cortex, with denser innervation in frontal and parietal regions compared to auditory and visual cortical areas. Superficial cortical layers receive more cholinergic input, while deeper layers rely on choline transporters for sustained acetylcholine production (Coppola and Disney, 2018). In addition to extrinsic cholinergic projections, local cholinergic interneurons (Goldberg et al., 2012) exist. Importantly, in the striatum, these interneurons are pivotal in modulating striatal activity (Goldberg et al., 2012).

A deeper understanding of how muscarinic receptors impact at the cellular, physiological and network level activity within and across these neural systems is key to understanding the therapeutic potential of muscarinic agents (McCutcheon et al., 2024).

**Figure 1.**
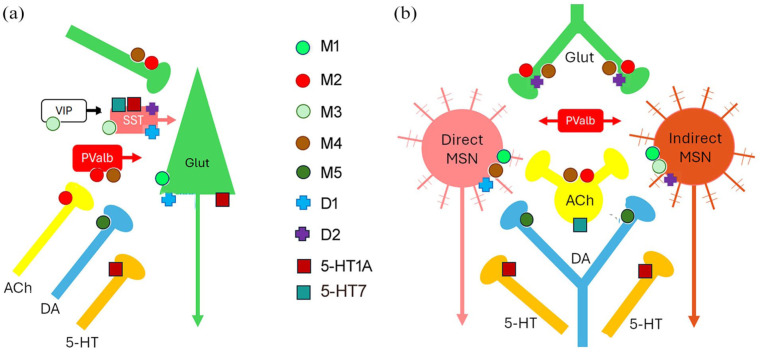
Schematic diagram showing most prominent locations of muscarinic receptors, along with D1, D2, 5-HT1A and 5-HT7 receptors, in prefrontal cortex (a) and striatum (b). MSN: medium spiny neuron; SST: somatostatin; Pvalb: parvalbumin; VIP: vasoactive intestinal polypeptide; Glut: glutamate.

Here, we summarise the location and functional role of muscarinic receptor subtypes across regions of CSTC loops, the amygdala and hippocampus in relation to RDoC domains. Figure 1 and Table 2 illustrate the cellular location of these receptor subtypes within the identified networks.

**Table 2.** Location of muscarinic receptor subtypes in corticostriato-thalamo-cortical circuitry, amygdala and hippocampus relevant to RDocC domains.

Region	Muscarinic receptor location
Cortex	M1 – pyramidal cell bodies
	M2 and M4 – thalamocortical glutamatergic terminals
	M2 and M4 – parvalbumin containing GABAergic interneurons
	M2 – cholinergic terminals from basal forebrain
Thalamus (auditory/visual)	M1 and M3 – thalamocortical glutamatergic cells
	M2 – GABAergic interneurons
Reticular nucleus of thalamus	M2 – GABAergic neurons
Striatum	M1 – GABAergic medium spiny neurons (D1 and D2)
	M2 and M4 – corticostriatal glutamatergic terminals
	M2 and M4 – cholinergic interneurons
	M4 – D1 containing medium spiny neurons
	M5 – dopamine terminals
Amygdala	M1 – pyramidal cell bodies
	M2 – interneurons
	M3 – glutamate and GABA terminals (heteroreceptors)
Hippocampus	M1 – pyramidal cell bodies (CA1-CA3) and dentate gyrus
	M2 – cholinergic nerve terminals (autoreceptor)
	M2 – glutamate and GABA terminals (heteroreceptors)
	M3 – glutamate terminals (heteroreceptor)
	M4 – association and commissural pathways

## Cortex

M1 receptors are located postsynaptically to cholinergic afferents on glutamatergic pyramidal cells (Yi et al., 2014), whereas M2 receptors are located predominantly in presynaptic axons (cholinergic and glutamatergic terminals) and to a lesser extent postynaptically (Mrzljak et al., 1993; Tsolias and Medalla, 2021). M2 muscarinic receptors are commonly found as inhibitory presynaptic autoreceptors (Figure 1), and so muscarinic antagonists blocking these receptors elevate Ach release, for example in prefrontal cortex, nucleus accumbens and striatum (Toide and Arima, 1989).

The role of M1 receptors in modulating excitatory/inhibitory balance between pyramidal cells and GABAergic interneurons has been proposed to play a role in cognition as well as ‘psychosis’. One perspective suggests that M1-mediated excitation of GABAergic interneurons that synapse onto pyramidal neurons leads to reduced cortical output to the striatum (Paul et al., 2022). By contrast, other evidence indicates that M1 receptors activation directly excites pyramidal neurons in the prefrontal cortex, and that this pyramidal cell activity indirectly increases the firing of parvalbumin expressing interneurons (Tikhonova et al., 2018). These contrasting findings highlight that the precise cellular mechanisms through which M1 receptors modulate prefrontal cortex activity remain incompletely understood. In addition, the effects of M1, M2 and M4 receptor activation on cortico-cortical communication, as well as on the output of cortical projections to other brain regions including the hippocampus and amygdala, should be considered.

## Hippocampus

In the septohippocampal pathway, M2 receptors function as autoreceptors on cholinergic nerve terminals, inhibiting ACh release. In the perforant pathway, which provides excitatory input to the dentate gyrus from the entorhinal cortex, M1 receptors are postsynaptic, while M2, M3 and M4 receptors modulate glutamate release as heteroreceptors. M2 heteroreceptors are found on GABAergic neurons in the association pathway, whereas M4 receptors are presynaptic on both association and commissural pathways (Levey et al., 1995; Rouse et al., 1999).

The presence of presynaptic muscarinic receptors regulating glutamate and GABA release in these pathways may be crucial for memory formation and retention. CA1 pyramidal cells, the primary glutamatergic output cells of the hippocampus, express M1 and to a lesser extent, M3 receptors. Notably, M1 receptors mediate ACh’s enhancement of N-methyl-D-aspartate (NMDA) receptor function, suggesting multiple pathways through which muscarinic receptors influence hippocampal function.

## Striatum

The striatum receives dense glutamatergic projections from widespread cortical and thalamic regions, integrating information relevant to action selection and decision-making. GABAergic medium spiny neurons (MSNs) and interneurons process this input, with giant aspiny cholinergic interneurons playing a particularly prominent role. Despite constituting only a small percentage of striatal neurons, these interneurons are tonically active and maintain basal ACh levels across the striatum (Contant et al., 1996; Goldberg et al., 2012). Their diverse activity patterns are associated with behaviours including reward processing and salience (Aosaki et al., 1994).

Within the striatum, M1 receptors are located on both D1 and D2 containing MSNs (Figure 1; Hersch et al., 1994; Ince et al., 1997; Paul et al., 2024) and hence activation of M1 receptors may modulate activity of both direct and indirect basal ganglia pathways. The dopaminergic neurons in the substantial nigra pars compacta and VTA express M5 muscarinic receptors on their cell soma and terminals in the dorsal and ventral striatum (Kayadjanian et al., 1999; Kuroiwa et al., 2012; Weiner et al., 1990), while M4 receptors are most prominent on cholinergic interneurons and striatonigral direct projection cells (Hersch et al., 1994; Kayadjanian et al., 1999; Phan et al., 2024; Weiner et al., 1990; Figure 1).

Thus, mAChRs are appropriately located to exert a powerful influence on striatal function in terms of both input (including dopaminergic input) and output. Taken together, these data support an involvement of all muscarinic receptor subtypes in corticostriatal processing of relevance to RDoC domains of cognitive systems (prediction error dysfunction) and positive valence systems (reward sensitivity and learning).

## Thalamus

Muscarinic receptor expression in the thalamus is of relatively low abundance compared to the high abundance of M1 and M4 receptors in the cortex and striatum (Buckley et al., 1988; Wall et al., 1991). Nevertheless, M1, M2, M3 and M4 receptors are present across thalamic regions and show important functional roles (Dean, 2004; Plummer et al., 1999; Zhu and Heggelund, 2001). One crucial role of the thalamus is to relay sensory information from the periphery to the cortex in a state-dependent manner (Sherman and Guillery, 2009). Muscarinic M2 receptors have been demonstrated in several components of the central auditory pathway, including cochlear nucleus, inferior colliculus, medial geniculate nucleus of the thalamus and auditory cortex (Hamada et al., 2010). In studies of the lateral geniculate nucleus, M1 and M3 receptors are present in the cell bodies and dendrites of thalamocortical relay cells, and M2 receptors in the cell bodies and dendrites of interneurons. Importantly, muscarinic M2 receptor activation of GABAergic interneurons in visual thalamic nuclei switched their firing pattern from bursting to tonic, strongly influencing patterns of thalamocortical neuronal activity (Zhu and Heggelund, 2001). Taken together, these data highlight the importance of sensory thalamic nuclei in perception of auditory and visual stimuli and a role for M1–M3 receptors in the RDoC sensorimotor domain.

It should be noted that cellular localisation studies of muscarinic receptor expression in other thalamic nuclei are relatively sparse. For example, although the mediodorsal thalamus expresses M1 and M4 receptors (Dean et al., 2004), the cellular localisation of these receptors is unclear. Mediodorsal thalamic connectivity to the PFC is associated with working memory, and hence modulation of muscarinic receptor activity in mediodorsal-prefrontal circuits could impact the RDoC working memory construct.

In summary, there is widespread expression of distinct muscarinic receptor subtypes across CSTC loops (see Table 2). Importantly, these loops map onto distinct symptom clusters that transcend traditional diagnostic categories, aligning with major RDoC constructs such as positive and negative valence systems and cognitive systems. Understanding how activation of specific muscarinic receptors alters neural activity within these loops to modify behaviour is crucial for linking muscarinic function to RDoC domains relevant to schizophrenia.

One largely overlooked mechanism is the regulation of CSTC loop circuits by the thalamic reticular nucleus (TRN). The TRN is a thin layer of GABAergic neurons surrounding other thalamic nuclei. It receives collateral glutamatergic inputs from both corticothalamic and thalamocortical projections but does not directly project to the cortex. Instead, the TRN sends dense inhibitory outputs to other thalamic nuclei, positioning it as a key modulator of corticothalamic communication (Pratt and Morris, 2015; Steriade et al., 1997).

Distinct TRN sectors represent different modalities, including sensory, motor, limbic, and cognitive functions, and hence are conceivably relevant to RDoc domains of sensorimotor, positive valence, cognitive systems, arousal and regulation, and social processes.

The importance of the TRN in regulating sensorimotor processes involving auditory structures (Table 1) conceivably relates to auditory hallucinations.

Although some overlap exists, specific TRN regions are linked to specific thalamic nuclei, such as the anterior ventral TRN with the mediodorsal thalamus for cognition. Notably, PFC and mediodorsal thalamus collaterals terminate more extensively in the TRN than those from other cortical and thalamic regions, suggesting that PFC circuits play a crucial role in gating thalamic relay activity. This is particularly relevant to RDoC cognitive systems constructs of executive function and working memory.

Inputs from the amygdala indicate the TRN’s involvement in assigning emotional salience to sensory filtering (Zikopoulos and Barbas, 2007, 2012). This is relevant to RDoC constructs of ‘arousal and regulation’ and ‘social processes’ involved in the blunted affect and associability elements of negative symptoms of schizophrenia.

TRN neurons exhibit two firing modes: high-frequency burst firing and tonic firing. Low-voltage-activated T-type calcium channels enable rhythmic burst firing, which is essential for generating thalamocortical oscillations. The TRN contributes to various brain rhythms, including sleep spindles, slow oscillations, and delta and gamma rhythms (Huguenard and McCormick, 2007; Macdonald et al., 1998). Importantly, changes in delta and gamma rhythms are implicated in cognition and perceptual changes in schizophrenia. For example, changes in delta auditory responses have been associated with auditory perceptual changes (hallucinations) and verbal working memory changes in schizophrenia (Puvvada et al., 2018). There is a robust literature on abnormal gamma band activity in schizophrenia relevant to RDoC domains of cognition and perception (Barch and Young, 2023; McNally and McCarley, 2016; Williams and Boksa, 2010). Muscarinic antagonism has been linked to the ability to enhance gamma oscillatory power (Pinault and Deschênes, 1992), as indeed has 5-HT7 antagonism (Leiser et al., 2014). M4 receptors seem to be important in the modulation of gamma oscillations (Gould et al., 2016), although the locus of action is unclear, while M2 receptors play an important role in modulating TRN function (Beierlein, 2014; Sun et al., 2013). Hence, muscarinic agonists or antagonists could conceivably act to modify multiple RDoC domains important in schizophrenia.

It may be relevant that, in contrast to the general low abundance of mAChR expression in thalamus, high levels of 5-HT7 receptors are located in specific thalamic nuclei, including centrolateral, centromedian and paraventricular nuclei (Gustafson et al., 1996). These nuclei are closely connected to corticostriatal loops, both motor and limbic (Kirouac, 2025; Sadikot and Rymar, 2009; Sato et al., 1979), and also play a role in modulating attention to auditory stimuli (Alanazi et al., 2024). This raises the possibility that their location at potentially important circuit hubs could be exploited clinically. Our research at Psychiatric Research Institute of Neuroscience in Glasgow, provided early evidence that an agent – which we termed ‘serominic’ – combining M4 muscarinic partial agonist activity with 5-HT7 receptor antagonist activity, without D2 receptor antagonist activity, might offer synergistic therapeutic benefits for the treatment of schizophrenia (Suckling et al., 2007).

Studies in our laboratory have shown the TRN to be a hub region of brain networks altered in genetic and NMDA receptor antagonist models relevant to schizophrenia (Dawson et al., 2012, 2015). Restoration of these network deficits through modification of TRN activity could potentially improve many symptoms of schizophrenia. The action of xanomeline at TRN M2 and possibly M4 receptors could potentially contribute to its therapeutic effects, as could an action at TRN 5-HT1A and 5-HT7 receptors.

## Clinical trials

Considering the similarities between xanomeline and clozapine in terms of muscarinic and 5-HT1A receptor effects, it is useful to consider the extensive clinical experience with clozapine briefly, before turning to the recent and as yet limited experience with xanomeline. As compared to the majority of drugs used to treat schizophrenia, active only against the positive symptoms, clozapine is unusual in that it may have some slight ability to alleviate the negative and cognitive symptoms that are resistant to most drugs (Huhn et al., 2019; Meyer-Lindenberg et al., 1997; Murphy et al., 2006; Nakajima et al., 2015; Siskind et al., 2016). This uncharacteristic property, which admittedly is small in comparison to the degree of impairment, seems to be shared by two other drugs, amisulpride and zotepine (Boyer et al., 1995; Danion et al., 1999; Huhn et al., 2019; Krause et al., 2018; Loo et al., 1997; Meyer-Lindenberg et al., 1997; Murphy et al., 2006). It is interesting to note that these two drugs share clozapine’s inverse agonist action at 5-HT7 receptors, but lack muscarinic receptor affinity (Abbas et al., 2009; Jahreis et al., 2023; Leopoldo et al., 2011; Purohit et al., 2005; Schotte et al., 1996).

Where clozapine clearly separates from other existing drugs is in its efficacy against positive symptoms in some of the ~30% of patients who show no benefit to any other anti-schizophrenia drug (Elkis and Buckley, 2016; Jauhar et al., 2022; Kane et al., 1988; Morrison et al., 2025; Siskind et al., 2016; Wagner et al., 2021). Which part of clozapine’s rich pharmacological profile mediates this action is not known. If muscarinic receptor partial agonism is involved, then it is conceivable that new drugs such as xanomeline could also be effective in some non-responders.

Side effects of clozapine are most prominently weight gain (likely mediated by H1 histamine receptor antagonism; Kim et al., 2007), and cholinergic partial agonist effects including hypersalivation, nausea and diarrhoea (Kontaxakis et al., 2005; Safferman et al., 1991; Young et al., 1998).

For xanomeline, recent results from 5-week placebo-controlled trials have been published. The results have been reviewed extensively elsewhere (Fabiano et al., 2024; Javitt, 2025; McKenna et al., 2024). Xanomeline was combined with a broad-spectrum muscarinic antagonist unable to penetrate into the brain (trospium) to combat the peripheral cholinergic side effects of xanomeline. This was broadly successful. Administration of the combination, KarXT, produced some cholinergic and some anti-cholinergic side-effects. As was probably predicted, the addition of trospium reduced the peripherally mediated partial agonist cholinergic side effects of xanomeline (hypersalivation and diarrhoea) without reducing those due to peripherally-mediated antagonist actions (tachycardia due to M2 antagonism) or centrally mediated partial agonist actions (nausea) (Alt et al., 2016; Beleslin and Nedelkovski, 1988; Brannan et al., 2021; Correll et al., 2022; Sramek et al., 1995). Remaining side effects are reported as mainly mild and diminishing over time.

In terms of efficacy, xanomeline is reported as reducing both positive and negative symptoms with greater benefit at 5 weeks as compared to 2 weeks (Brannan et al., 2021; Correll et al., 2022; Horan et al., 2024; Kaul et al., 2024, 2025). This quite slow onset of efficacy against positive symptoms reproduces experience with existing D2 dopamine antagonist drugs and could be interpreted tentatively as evidence for some commonality of action. Some benefit against cognitive symptoms was also observed, although sample sizes were low for these to be reliably detected (Horan et al., 2025). Preliminary reports yet to be fully published confirmed long-term safety and efficacy in 52-week trials (Smith et al., 2025).

Published results for emraclidine are as yet even more limited, but in a small phase 1b trial, it also showed the ability to attenuate both positive and negative symptoms (Krystal et al., 2022), with beneficial effects also increasing gradually over 6 weeks. There were fewer side effects reported compared to KarXT, matching emraclidine’s more restricted range of target receptors. However, two recent Phase II clinical trials in schizophrenia were disappointing in that emraclidine did not produce beneficial effects on positive and negative symptoms. In these studies, the placebo response was high, which may have been a contributing factor in the outcome: https://www.biospace.com/drug-development/abbvie-shares-plummet-12-as-cerevel-schizophrenia-asset-fails-phase-ii-trials. In parallel, Neurocrine Biosciences is conducting clinical trials of muscarinic agents for schizophrenia, with results awaited with anticipation.

## Summary and conclusion

Muscarinic receptor subtypes are abundant in neural systems relevant to multiple RDoC domains and hence have the potential to improve positive, negative and cognitive symptoms in schizophrenia. Evidence to date suggests that partial agonist activity at M1 and/or M4 receptors could offer a viable strategy to treat aspects of schizophrenia. However, in the case of xanomeline/KarXT, actions at other muscarinic receptors (M2, M3 and M5) and also at serotonin receptors will contribute to its actions in the CNS. The relative contribution of each subtype, and whether agonism or partial agonism is important, remains unclear. There are early suggestions that muscarinic partial agonist drugs could show beneficial activity against negative and cognitive symptoms, which, if confirmed, might offer advantages over existing drugs. Xanomeline shows an overlapping pharmacological profile to clozapine – partial agonist activity at M1 and M4 muscarinic receptors, and at 5-HT1A receptors, together with affinity for 5-HT7 receptors. By comparison, with existing antipsychotic drugs, xanomeline has very weak affinity for dopamine receptors. Since partial agonism at AChR M1/M4 receptors indirectly reduces dopamine transmission, additional blockade of dopamine receptors may be unnecessary. As clinical experience with KarXT grows, valuable insight into the mechanisms of action of these drugs will doubtless be revealed from a comparison of their therapeutic effects. The pharmacological similarities with clozapine also raise the possibility that KarXT might also show efficacy in some people who do not respond to existing drugs.

In summary, there is certainly cause for optimism that these emerging drugs will find a place in the therapeutic options available to treat the symptoms of schizophrenia. While muscarinic agonism/partial agonism holds promise, we propose that a multi-targeted approach combining actions at 5-HT1A and 5-HT7 receptors may provide additional therapeutic benefits across a wide range of RDoC domains. This strategy could therefore offer therapeutic advantages not only for schizophrenia but also transdiagnostically for other neuropsychiatric conditions

## Can muscarinic drugs make a fundamental difference?

The anatomical location and functional involvement of specific muscarinic receptors across multiple CSTC loops and related networks provide a strong rationale for the potential of muscarinic drugs to make a fundamental difference in the treatment of schizophrenia. By targeting a wide range of RDoC domains, these drugs may address the full spectrum of symptoms (positive, negative and cognitive), offering a more comprehensive approach than current dopamine receptor antagonists, which primarily alleviate hallucinations and delusions, but have limited impact on negative symptoms and cognitive deficits.

A key distinction between muscarinic receptor modulation and existing dopamine-based antipsychotic drugs is the reduced likelihood of dopamine-mediated side effects. Dopamine is involved in diverse brain functions, including movement control, cognition, reward-motivated behaviour, metabolic processes and lactation. Unsurprisingly, blocking dopamine receptors in systems involved in these functions can lead to a wide range of adverse effects, such as abnormal movement (extrapyramidal symptoms), altered motivation, metabolic disturbances and sedation. Additionally, weight gain – likely mediated through H1 histamine receptor antagonism – is a common side effect of several existing antipsychotic drugs which block both H1 and D2 receptors. Collectively, these side effects can negatively impact physical health, overall quality of life and integration in society. Hence, muscarinic drugs may offer a fundamental advantage by minimising these burdensome side effects.

However, muscarinic agents such as xanomeline are not without side effects, including nausea and peripherally mediated cholinergic side effects (hypersalivation and diarrhoea). These can be reduced by co-administration with trospium, a peripherally acting broad-spectrum muscarinic antagonist. However, trospiums’s M2 receptor antagonism may lead to tachycardia. The development of compounds that selectively target central muscarinic receptors represents an important next step, as this would minimise peripherally mediated adverse effects.

Psychiatry is increasingly moving towards more personalised and comprehensive approaches. In this context, the emergence of muscarinic agents for schizophrenia, with the potential to improve the full spectrum of symptoms and to offer a more favourable side-effect profile than traditional dopamine-based antipsychotic drugs, could represent a long-awaited shift in therapeutic strategy.

In conclusion, based on neurobiological evidence and emerging clinical findings, there is considerable reason for optimism that muscarinic receptor modulation (with additional 5-HT receptor modulation) has the potential to make a fundamental difference not only in the treatment of schizophrenia but also in other neuropsychiatric and neurodegenerative conditions that share disruptions across similar RDoC domains.
